# Hyperbilirubinemia in Preterm Infants Admitted to Neonatal Intensive Care Units in Ethiopia

**DOI:** 10.1177/2333794X20985809

**Published:** 2020-12-28

**Authors:** Sara Aynalem, Mahlet Abayneh, Gesit Metaferia, Abayneh G. Demissie, Netsanet Workneh Gidi, Asrat G. Demtse, Hailu Berta, Bogale Worku, Assaye K. Nigussie, Amha Mekasha, Zelalem Tazu Bonger, Elizabeth M. McClure, Robert L. Goldenberg, Lulu M. Muhe

**Affiliations:** 1 St. Paul’s Hospital Millennium Medical College, Addis Ababa, Ethiopia; 2 University of Gondar, Gondar, Ethiopia; 3 Jimma University, Jimma, Ethiopia; 4 Addis Ababa University, Addis Ababa, Ethiopia; 5 Zewditu Hospital, Addis Ababa, Ethiopia; 6 Ethiopian Pediatric Society, Addis Ababa, Ethiopia; 7 Bill and Melinda Gates Foundation, Seattle, WA, USA; 8 RTI International, Durham, NC, USA; 9 Columbia University, New York, NY, USA

**Keywords:** prematurity, neonatal hyperbilirubinemia, acute bilirubin encephalopathy

## Abstract

*Background*. Hyperbilirubinemia is prevalent and protracted in preterm infants. This study assessed the pattern of hyperbilirubinemia in preterm infants in Ethiopia. *Methods*. This study was part of multi-centered prospective, cross-sectional, observational study that determined causes of death among preterm infants. Jaundice was first identified based on clinical visual assessment. Venous blood was then sent for total and direct serum bilirubin level measurements. For this study, a total serum bilirubin level ≥5 mg/dL was taken as the cutoff point to diagnose hyperbilirubinemia. Based on the bilirubin level and clinical findings, the final diagnoses of hyperbilirubinemia and associated complications were made by the physician. *Result*. A total of 4919 preterm infants were enrolled into the overall study, and 3852 were admitted to one of the study’s newborn intensive care units. Of these, 1779 (46.2%) infants were diagnosed with hyperbilirubinemia. Ten of these (0.6%) developed acute bilirubin encephalopathy. The prevalence of hyperbilirubinemia was 66.7% among the infants who were less than 28 weeks of gestation who survived. Rh incompatibility (*P* = .002), ABO incompatibility (*P* = .0001), and sepsis (*P* = .0001) were significantly associated with hyperbilirubinemia. Perinatal asphyxia (*P*-value = 0.0001) was negatively associated with hyperbilirubinemia. *Conclusion.* The prevalence of hyperbilirubinemia in preterm babies admitted to neonatal care units in Ethiopia was high. The major risk factors associated with hyperbilirubinemia in preterm babies in this study were found to be ABO incompatibility, sepsis, and Rh isoimmunization.

## Background

Globally, preterm complications are among the most common causes of neonatal death. Hyperbilirubinemia is one of the preterm complications known to contribute to neonatal mortality. Almost all newborn infants have a total serum or plasma bilirubin (TSB) level greater than 1 mg/dL (17 µmol/L), which is the upper limit of normal value for adults. As the total serum bilirubin level increases beyond 5 mg/dL, it will start manifesting clinically as neonatal jaundice. Jaundice is the yellowish discoloration of the skin and/or conjunctiva caused by bilirubin deposition.^1^ Neonatal jaundice is a very common condition worldwide, occurring in up to 60% of term and 80% of preterm newborns in the first week of life.^2^

Hyperbilirubinemia in preterm infants is more prevalent, severe, and protracted than in term infants due to the short life span of their red blood cells (RBCs), and the immaturity of their liver and gastrointestinal tracts. Often, there is also a delay in enteral feeding, which may limit intestinal motility and bacterial colonization, resulting in decreased clearance of bilirubin. These developmental and clinical phenomena contribute to the greater degree and duration of neonatal hyperbilirubinemia in premature infants.^3^ One of the major complications of an elevated TSB level is acute bilirubin encephalopathy (ABE), which occurs when circulating bilirubin crosses the blood-brain barrier, binds to brain tissue, and ultimately causes a spectrum of neurologic problems. Surviving infants may acquire long-term neurodevelopmental sequelae such as cerebral palsy, sensorineural hearing loss, intellectual difficulties or gross developmental delays.^4^

Available clinical guidelines note that early detection and intervention of infants at risk of severe hyperbilirubinemia can facilitate timely and effective prevention of the associated complications.^1^ Currently, available evidence suggests that low- and middle-income countries disproportionately bear the maximum burden of severe neonatal hyperbilirubinemia.^5^ A study done in Addis Ababa found that ABO incompatibility and sepsis were among the risk factors for the occurrence of hyperbilirubinemia.^6^ A study done in West India University in 2012 indicated that ABO incompatibility and Rh incompatibility were associated with hyperbilirubinemia.^7^ Also, another study done in Benin showed ABO incompatibility and sepsis were considered as risk factors for hyperbilirubinemia.^8^ A systematic review of hyperbilirubinemia in low-resource countries by Slusher et al showed that birth trauma was associated with neonatal hyperbilirubinemia.^9^ Therefore, early detection of hyperbilirubinemia in at-risk infants is very important for prevention of complications.

Although the United Nations’ Millennium Develop-ment Goal (MDG) and Sustainable Development Goals (SDG) initiatives have not given due attention to hyperbilirubinemia, hemolytic diseases of the newborn and other causes of neonatal hyperbilirubinemia are increasingly acknowledged as an important contributor to global neonatal morbidity and mortality.^10^ Since preterm babies are more likely to develop severe hyperbilirubinemia compared to term neonates, this research aims to assess the prevalence, associated risk factors and outcome of hyperbilirubinemia in preterm newborns in 5 tertiary hospitals of Ethiopia.

### General Objectives

Our main objective was to assess the pattern of hyperbilirubinemia in preterm neonates in 5 Hospitals of Ethiopia.

Specific objectives:

- To assess the prevalence of hyperbilirubinemia in preterm infants admitted to 5 newborn intensive care units (NICU).
- To identify the associated risk factors for hyperbilirubinemia among preterm neonates admitted to an NICU.
- To determine the prevalence of acute bilirubin encephalopathy in preterm neonates who were diagnosed with hyperbilirubinemia.

### Study Setting and Design

This study was part of a multicenter, prospective, cross-sectional, observational clinical study done in 5 hospitals in Ethiopia over a period of nearly 2 years (from July 1, 2016, to May 31, 2018). The detailed protocol has been published.^11^ This study was primarily done to identify major causes of death among preterm babies in 5 hospitals in Ethiopia (Gondar University Hospital, Jimma University Hospital, St. Paul’s Hospital Millennium Medical College, Ghandi Memorial Hospital and Tikur Anbessa Hospital). Ghandi Memorial Hospital participated over a period of 7 months (from July 1, 2016, to January 31, 2017). A total of 4919 preterm babies and their mothers participated in the study, among the total of 7368 babies who were assessed for eligibility. From the main study, 3852 neonates who were admitted to an NICU were enrolled. This supplementary study aimed to assess the prevalence of hyperbilirubinemia, associated risk factors, and the prevalence of acute bilirubin encephalopathy among preterm neonates admitted to NICUs.

The study participants were preterm infants born at a study hospital or who were referred within 7 days of life to 1 of the 5 hospitals. Gestational age was determined by using a hierarchy of 3 methods: ultrasound before 28 weeks of gestation when available, the mother’s report of her last menstrual period when judged reliable and the New Ballard Score. Data were collected on socioeconomic status, obstetric history, clinical condition, and laboratory and imaging studies. Jaundice was first identified based on visual assessment of the infant and then 2.5 to 3.0 mL of venous blood was sent to the laboratory for measurement of the total serum bilirubin and direct bilirubin levels.

Although several TSB measurements may have been done for each preterm baby suspected of having hyperbilirubinemia, for this study, a TSB of more than 5 mg/dL in any sample was used as the cutoff point to diagnose hyperbilirubinemia. Based on the TSB level, the clinical findings of hyperbilirubinemia, the final diagnosis of hyperbilirubinemia, and its complications were made by the treating physician. We evaluated the available medical records to define the clinical characteristics that were potential risk factors for hyperbilirubinemia, including Rh incompatibility, ABO incompatibility, hemorrhagic disease, head trauma during birth, sepsis, feeding problem, birth asphyxia, polycythemia, and hypoglycemia. Even though, Glucose-6-phosphate dehydrogenase enzyme deficiency is one of the major cause of neonatal hyperbilirubinemia, it is not included in this study as a potential risk factor because G-6-PD enzyme assay is not done for any of the babies in this study, as neonatal screening for the enzyme deficiency is not routinely carried out in our country.

The final diagnosis made by the treating physician was used. The clinical management of patients followed the national guideline developed by federal ministry of health of Ethiopia.^12^ For Neonates less than 35 weeks we used guidelines suggested by Maisels et al^13^ as indications for phototherapy and exchange transfusion. Acute bilirubin encephalopathy (ABE) was diagnosed based on clinical characteristics which include 3 phases. Phase-I considered in the first 2 days of age with poor motor reflex, high pitched cry, decreased tone, lethargy, and poor feeding. Phase-II is considered in the middle of the first week with hypertonia, seizure and depressed sensorium, fever, opisthotonos posturing, paralysis of upward gazing. Phase-III is considered after 1 week of age and the patient has decreased hypertonia, hearing and visual abnormality, poor feeding, athetosis, and seizure.^12^

## Eligibility

All preterm live-born infants who were admitted to one of the study hospitals with a gestational age of less than 37 completed weeks were potentially eligible. The study hospital staff recruited preterm infants who were alive without any lower limit of gestational age. The following were the study inclusion criteria:

Either delivered at or the baby transferred to one of the participating study hospitals; gestational age was < 37 weeks according to the algorithm using the 3 methods; live-born was defined as cry, breathing and/or movement after delivery or Apgar ≥ 1; infant age was < 7 days when screened, and consent was given for study participation.

Exclusion criteria included a gestational age that could not be reliably determined using the study criteria. All eligible live-born babies who met the gestational age criteria were enrolled in the study regardless of whether the baby died prior to admission to the NICU or was discharged home without admission.

## Quality Assurance

All preterm infants in the study hospital NICUs were evaluated twice daily by a research nurse who documented the findings on case report forms. A supervisor checked that all forms were complete, the sample for laboratory evaluation was taken in sufficient quantity and quality and relevant laboratory, radiology or pathology personnel were notified of the sample sent. The investigators conducted site visits to each hospital routinely to check data quality and provide support as necessary.

## Data Entry and Statistical Analysis

Data were entered twice into the computer using the data management system developed for the study and were transferred on a weekly basis from each data management computer to the data center at Addis Ababa University. This created a complete data repository where it was merged to 1 master data set for analysis. Data were coded, entered and analyzed using SPSS version 20. Descriptive statistics with frequency and percentages, tables, and cross-tabulations were used. Binary logistic regression statistical models were used for analysis and to determine the relationship between the dependent and the independent variables. *P*-values ≤ .05 were used to identify statistically associated factors.

## Ethical Approval and Informed Consent

The study was conducted after ethical approval was obtained from Addis Ababa University College of Health Sciences Institutional Review Board (Ethics ID: AAUMF 03-008). Written consent for participation was obtained from the parent or legal guardian who enrolled in the study. Consent was obtained in English, Amharic, and Oromifa languages, as appropriate, and only data from the women and infants of women who provided informed consent were enrolled. Confidentiality of the information was maintained.

## Results

**Figure 1.**
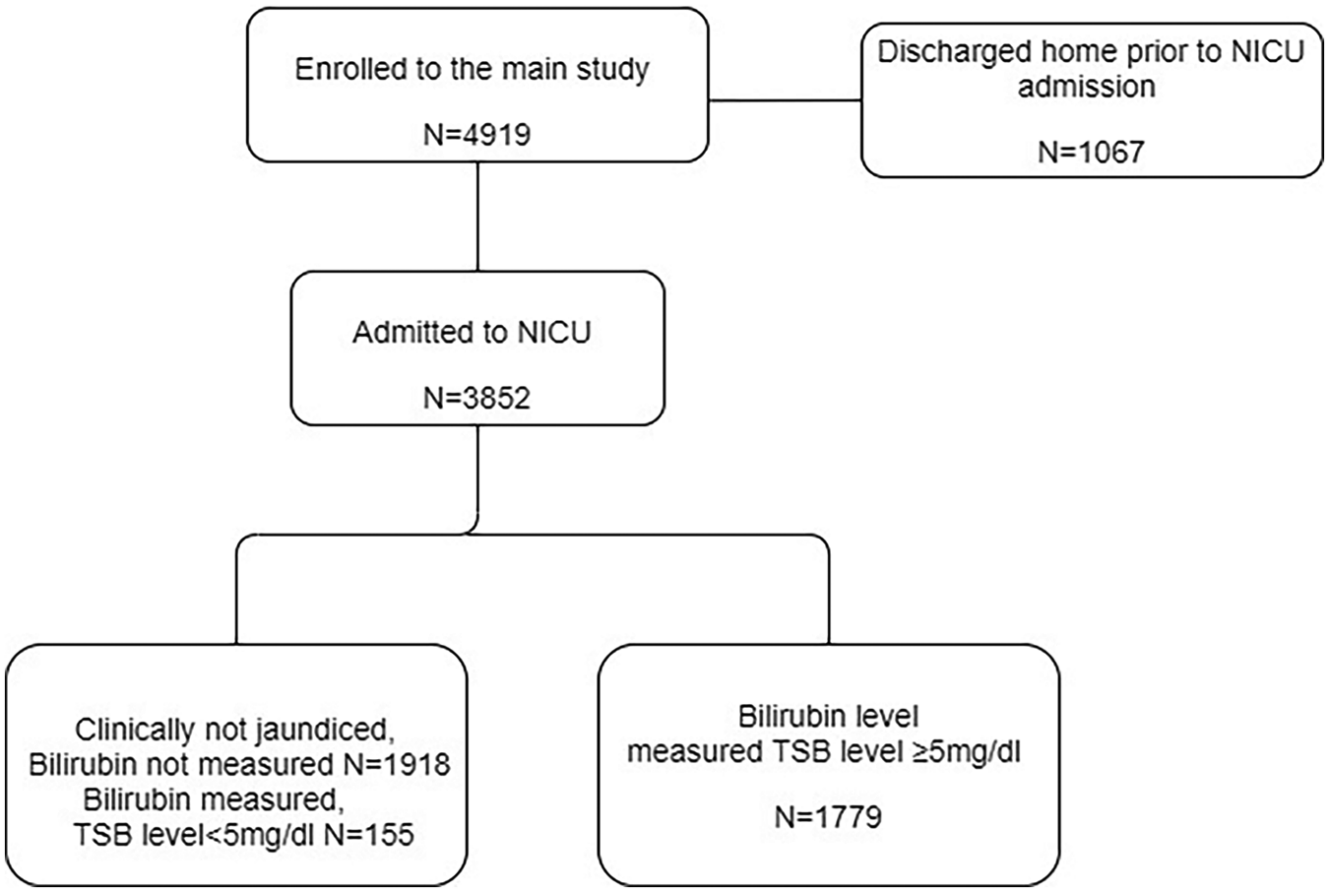
Enrollment flow diagram.

As shown in Figure 1, among the total of 3852 neonates admitted to an NICU, 1779 (46.2%) were diagnosed with hyperbilirubinemia. Out of these 3852 neonates, neonatal hyperbilirubinemia was detected in 966 (54.9%) male neonates and in 795 (45.1%) of female neonates. The difference between male and female infants on the prevalence of hyperbilirubinemia was not significant.

Table 1 describes the prevalence of hyperbilirubinemia in preterm neonates. About 89 out of the total 104 preterm neonates whose gestational age was less than 28 weeks died, usually soon after admission. Thus, these preterm babies died before their bilirubin level was measured. We, therefore, compared the prevalence of hyperbilirubinemia among preterm newborns who survived until discharge.

**Table 1.** Prevalence of Hyperbilirubinemia Among Preterm Neonates.

Gestational age category	Status	Total *N* all neonates *N* (%)	Neonates with hyperbilirubinemia *N* (%)	Neonates without hyperbilirubinemia *N* (%)
<28 weeks	All	104 (100)	25 (24.0)	79 (76.0)
	Alive	15 (100)	10 (66.7)	5 (33.3)
	Died	89 (100)	15 (16.9)	74 (83.1)
28 to 31 weeks	All	931 (100)	360 (38.7)	571 (61.3)
	Alive	391 (100)	221 (56.5)	170 (43.5)
	Died	540 (100)	139 (25.7)	401 (74.3)
32 to 34 weeks	All	1636 (100)	838 (51.2)	798 (48.8)
	Alive	1250 (100)	733 (58.6)	517 (41.4)
	Died	386 (100)	105 (27.2)	281 (72.8)
35 to <37 weeks	All	1181 (100)	556 (47.1)	625 (52.9)
	Alive	985 (100)	502 (51.0)	483 (49.0)
	Died	196 (100)	54 (27.6)	142 (72.4)

As shown in Table 1, out of the total 15 neonates whose gestational age was less than 28 weeks and survived, 10 (66.7%) developed hyperbilirubinemia. Among those whose gestational age was 28 to 31 weeks, 32 to 34 weeks, and 35 to <37 weeks, 221 (56.5%), 733 (58.6%), and 502 (51.0%) developed hyperbilirubinemia, respectively.

Among the total preterm neonates who developed hyperbilirubinemia, 25 were <28 weeks and of these, 10 (40.0%) survived and 15 (60.0%) died. Of the 360 neonates with hyperbilirubinemia whose gestation was 28 to 31 weeks, 221 (61.4%) survived and 139 (38.6%) died. Of the 838 neonates 32 to 34 weeks, 733 (87.5%) survived and 105 (12.5%) died. And of those 556 neonates between 35 and <37 weeks, 502 (90.1%) survived and 54 (9.7%) died.

Table 2 shows the bilirubin levels measured on preterm neonates who had hyperbilirubinemia. As the level of bilirubin increased, the mortality also increased among those with lower gestational ages (<28 weeks) compared to those with gestational ages of 28 to 31, 32 to 34, and 35 to <37 weeks. The level of bilirubin among those 1779 preterm neonates who had hyperbilirubinemia, 1324 (74.4%) had bilirubin levels within the range of 5 to 14 mg/dL. About 331 (18.6%) and 124 (7.0%) of the neonates had levels of 15 to 20 mg/dL and >20 mg/dL respectively.

**Table 2.** Pattern of Bilirubin Levels Measured in Preterm Neonates With Respect to Their Gestational Age and Their Outcome.

Gestational age (weeks)	Bilirubin level											
	<5 mg/dL			5 to 14 mg/dL			15 to 20 mg/dL			>20 mg/dL		
	All	Alive	Death *N* (%)	All	Alive	Death *N* (%)	All	Alive	Death *N* (%)	All	Alive	Death *N* (%)
<28	4	1	3 (75.0)	19	9	10 (52.6)	4	1	3 (75.0)	2	0	2 (100)
28 to 31	36	25	11 (30.6)	287	177	110 (39.1)	54	35	19 (35.2)	19	9	10 (52.6)
32 to 34	66	51	15 (22.7)	624	545	79 (12.7)	151	132	19 (12.6)	63	56	7 (11.1)
35 to <37	49	47	2 (4.1)	394	360	34 (8.6)	122	106	16 (13.1)	40	36	4 (10.0)

Table 3 summarizes the clinical characteristics of preterm newborns and their association with hyperbilirubinemia.

**Table 3.** Associated Risk Factors of Hyperbilirubinemia in Preterm Newborns.

Category	Neonates without hyperbilirubinemia *N* (%)	Neonates with hyperbilirubinemia *N* (%)	Odds ratio (adjusted) with 95% CI	*P*-value
Rh incompatibility				
Yes	11 (23.9)	35 (76.1)	3.34 (1.64-6.78)	
No	1974 (54.1)	1679 (45.9)	1	.002
ABO incompatibility				
Yes	19 (21.6)	69 (78.4)	4.18 (2.43-7.17)	
No	1963 (54.4)	1646 (45.6)	1	.0001
Hemorrhagic disease				
Yes	14 (58.3)	10 (41.7)	0.73 (0.31-1.69)	
No	1965 (53.7)	1696 (46.3)	1	.561
Head trauma during birth				
Yes	11 (44.0)	14 (56.0)	1.33 (0.57-3.10)	
No	2045 (53.7)	1760 (46.3)	1	.506
Sepsis				
Yes	724 (49.0)	755 (51.0)	1.31 (1.14-1.51)	
No	1246 (55.9)	981 (44.1)	1	.0001
Feeding problem				
Yes	536 (52.3)	488 (47.7)	1.04 (0.88-1.21)	
No	1480 (54.3)	1245 (45.7)	1	.737
Birth asphyxia				
Yes	169 (68.1)	79 (31.9)	0.52 (0.39-0.70)	
No	1822 (52.8)	1628 (47.2)	1	.0001
Polycythemia				
Yes	83 (54.2)	70 (45.8)	0.89 (0.63-1.25)	
No	1878 (52.9)	1669 (47.1)	1	.220
Hypoglycemia				
Yes	389 (53.1)	343 (46.9)	1.03 (0.86-1.22)	
No	1604 (54.0)	1365 (46.0)	1	.170

About 755 (51.0%), 343 (46.9%), and 69 (78.4%) of the infants had sepsis, hypoglycemia, and ABO incompatibility, respectively.

Neonatal hyperbilirubinemia was positively associated with several clinical characteristics such as Rh incompatibility OR 3.3 (95% CI 1.64-6.78, *P*-value = .002), ABO incompatibility OR 4.18 (95% CI 2.43-7.17, *P*-value = .0001), and sepsis (OR 1.31, 95% CI 1.14-1.51, *P*-value = .0001), and appeared negatively associated with perinatal asphyxia (OR 0.52, 95% CI 0.39-0.70, *P*-value = .0001) The other characteristics in the table were not significantly associated with the occurrence of neonatal hyperbilirubinemia (Table 3).

The mean serum bilirubin level of neonates with ABE was >15.5 mg/dL. Among the 10 preterm babies who developed ABE, in 7, their bilirubin level was above 20.1 mg/dL. In 2 of the patients, their bilirubin level was 15.5 mg/dL and 18.2 mg/dL, respectively. One neonate had a bilirubin level of 9.9 mg/dL and had more than 1 associated risk factor. The cause of hyperbilirubinemia was suspected sepsis with feeding problems for 6 infants, and 2 had respiratory distress syndrome as a diagnosis and the rest had asphyxia and Rh incompatibility as a cause of the hyperbilirubinemia. Out of the total of 10 infants who developed ABE, 7 of them died. The primary cause of death for those who developed ABE was sepsis (*N* = 4) and ABE (*N* = 1), asphyxia (*N* = 1), and IVH (*N* = 1).

## Discussion

Our study found that the prevalence of hyperbilirubinemia in preterm babies was 46.2%, of which less than 1% developed ABE. This finding shows that the prevalence of neonatal hyperbilirubinemia was higher than in prior studies conducted in 2 of the study hospitals (Tikur Anbesa hospital) which found a prevalence of 24.4%,^6^ and Gondar which found a prevalence of 24.6%.^14^ Studies from Nigeria have found a 32.5% prevalence,^15^ and 47.7% prevalence.^16^ One study from India showed a similar prevalence of 42%.^17^ A high-resource setting study conducted by Palmer and Drew^18^ in Australia showed a 20% prevalence.

Mortality among infants less than 28 weeks was very high and hyperbilirubinemia contributed to 11% of all deaths of preterm babies.^19^ Of the surviving preterm babies two-thirds developed hyperbilirubinemia. This finding is similar to the study done in India which found the highest prevalence among those less than 30 weeks.^17^ Another 2 studies from India also showed that infants with low gestational ages (<37 weeks) were at higher risk of severe hyperbilirubinemia.^20,21^

When we see the measured bilirubin levels, 7.0% of preterm infants had a level >20 mg/dL. As the bilirubin level increased, the highest mortality was seen in those infants younger than 28 weeks. In the era before the routine use of exchange transfusion and availability of phototherapy, Crosse et al found that 73.6% of preterm infants with kernicterus died as compared to 25.6% of all infants born prematurely. The highest mortality rate was among those infants with lower birth weights and the risk increased both as the gestational age decreased and as the concentration of total bilirubin rose.^22^ Another retrospective study done by Oh et al^23^ in infants with a birth weight less than 1000 g, found that total bilirubin concentrations during the first 14 days of birth were directly correlated with death, neurodevelopmental impairment, and sensorineural hearing loss. However, Morris et al highlighted that by comparing aggressive versus conservative phototherapy for extremely low birth weight infants (<500-750 g), suggests a tradeoff between reducing the risk of bilirubin induced neurologic dysfunction and death. The rate of neurodevelopmental impairment alone was significantly reduced with aggressive phototherapy, but the reduction offset by an increase in mortality among infants weighing 501 to 750 g at birth. This study also showed that neurodevelopmental outcomes were better with lower bilirubin levels while death was higher with lower bilirubin levels exposed to more aggressive phototherapy management.^24^ So although treatment of hyperbilirubinemia is still required to protect ELBW infants from bilirubin-induced neurologic dysfunction, even at lower levels of bilirubin because of compromised bilirubin-binding capacity in ELBW infants, especially in the first several days after birth, the type of treatment can affect the outcome.^25^

Our study showed that Rh incompatibility, ABO incompatibility, and sepsis were significantly associated with the occurrence of neonatal hyperbilirubinemia. Similar studies in Addis Ababa, Benin and in West India University found that ABO incompatibility, Rh incompatibility, and sepsis were associated with the occurrence of hyperbilirubinemia.^6-8^ Our study also found perinatal asphyxia was negatively associated with neonatal hyperbilirubinemia. This might be explained by the fact that acidosis in asphyxia is generally corrected soon after birth, before significant hyperbilirubinemia develops in preterm infants. Although 1 study from Pakistan showed birth asphyxia was a risk factor for severe jaundice.^26^ Another study done by Fekete et al^27^ found that perinatal asphyxia per se does not exaggerate hyperbilirubinemia either in full term or in preterm babies. Considering the above findings further research focusing more on this area might help identify why this results were different.

In this study, 10 babies (0.6%) developed acute bilirubin encephalopathy. In 1 study done in Calabar, South Nigeria, the prevalence of ABE was estimated at 0.7%,^28^ and another study in China showed a prevalence of 0.5% ABE in preterm neonates.^29^

Among the preterm babies who developed ABE, for the majority, the bilirubin level was above 20.1 mg/dL. Two had bilirubin levels less than 20 mg/dL. Of the neonates who developed ABE, most had sepsis and 1 patient had Rh incompatibility as a cause of hyperbilirubinemia. Two studies from Pakistan and Egypt indicated that infants with Rh disease were 20 times at increased risk of severe hyperbilirubinemia.^26,30^ Three reports from Pakistan, Egypt, and India indicated that infants diagnosed with sepsis were at increased risk of developing severe hyperbilirubinemia.^26,30,31^ These findings were similar to a finding on a systematic review that showed that LMICs consistently reported substantially higher rates of exchange transfusion and bilirubin-induced neurologic dysfunctions (ABE and chronic bilirubin encephalopathy or kernicterus) than in high-income countries.^9^

### Limitations

Our study had several limitations. First, different machines were used to determine TSB at the hospitals. Additionally, the procedures used to collect blood samples were not uniform across the study sites.

Finally, while different gestational ages have different cutoff points to diagnose hyperbilirubinemia, for the purpose of this study we used 5 mg/dL as a cut of point because we could not extract the information on the postnatal age that the blood was drawn.

## Conclusion and Recommendation

The prevalence of hyperbilirubinemia in hospital-admitted preterm babies was quite high. The major associated risk factors for hyperbilirubinemia in preterm babies in this study were found to be ABO incompatibility, sepsis, and Rh isoimmunization. Since neonatal hyperbilirubinemia may be associated with irreversible brain damage and a high level of mortality among preterm infants, routine screening and investigation for TSB are imperative for early detection and timely intervention.

## References

[1] Dennery PA Seidman DS Stevenson DK. Neonatal hyperbilirubinemia. N Engl J Med. 2001;344:581-590. 11207355 10.1056/NEJM200102223440807

[2] Slusher TM Angyo IA Bode-Thomas F, et al Transcutaneous bilirubin measurements and serum total bilirubin levels in indigenous African infants. Pediatrics. 2004;113:1636-1641. 15173484 10.1542/peds.113.6.1636

[3] Maisels MJ Bhutani VK Bogen D Newman TB Stark AR Watchko JF. Hyperbilirubinemia in the newborn infant ≥35 weeks’ gestation: an update with clarifications. Pediatrics. 2009;124:1193-1198. 19786452 10.1542/peds.2009-0329

[4] Mwaniki MK Atieno M Lawn JE Newton CR. Long-term neurodevelopmental outcomes after intrauterine and neonatal insults: a systematic review. Lancet. 2012;379:445-452. 22244654 10.1016/S0140-6736(11)61577-8 PMC3273721

[5] Bhutani VK Zipursky A Blencowe H, et al Neonatal hyperbilirubinemia and Rhesus disease of the newborn: incidence and impairment estimates for 2010 at regional and global levels. Pediatr Res. 2013;74:86-100. 24366465 10.1038/pr.2013.208 PMC3873706

[6] Kassa RT Gudeta H Assen ZM Mulugeta DT Teshome GS. Neonatal hyperbilirubinemia: magnitude and associated etiologic factors among neonates admitted at Tikur Anbessa specialized hospital, Ethiopia. J Pregnancy Child Health. 2018;5:2.

[7] Henny-Harry C Trotman H. Epidemiology of neonatal jaundice at the University Hospital of the West Indies. West Indian Med J. 2012;61:37-42. 22808564

[8] Israel-Aina Y Omoigberale A. Risk factors for neonatal jaundice in babies presenting at the University of Benin Teaching Hospital, Benin City. Niger J Pediatr. 2012;39:159-163.

[9] Slusher TM Zamora TG Appiah D, et al Burden of severe neonatal jaundice: a systematic review and meta-analysis. BMJ Paediatr Open. 2017;1:e000105. 10.1136/bmjpo-2017-000105 PMC5862199 29637134

[10] GBD Mortality and Causes of Death Collaborators. Global, regional, and national age–sex specific all-cause and cause-specific mortality for 240 causes of death, 1990–2013: a systematic analysis for the Global Burden of Disease Study 2013. Lancet. 2015;385:117-171. 25530442 10.1016/S0140-6736(14)61682-2 PMC4340604

[11] Muhe LM McClure EM Mekasha A, et al A prospective study of causes of illness and death in preterm infants in Ethiopia: the SIP Study Protocol. Reprod Health. 2018;15:116. 29945680 10.1186/s12978-018-0555-y PMC6020308

[12] Federal Ministry of Health of Ethiopia. Neonatal Intensive Care Unit (NICU) Management Protocol. Federal Ministry of Health of Ethiopia; 2014.

[13] Maisels MJ Watchko JF Bhutani VK Stevenson DK. An approach to the management of hyperbilirubinemia in the preterm infant less than 35 weeks of gestation. J Perinatol. 2012;32:660-664. 22678141 10.1038/jp.2012.71

[14] Yismaw AE Tarekegn AA. Proportion and factors of death among preterm neonates admitted in University of Gondar comprehensive specialized hospital neonatal intensive care unit, Northwest Ethiopia. BMC Res Notes. 2018;11:867. 30522518 10.1186/s13104-018-3970-9 PMC6282301

[15] Onyearugha CN Onyire BN Ugboma HAA Neonatal jaundice: prevalence and associated factors as seen in Federal Medical Centre Abakaliki, Southeast Nigeria. J Clin Med Res. 2011;3:40-45.

[16] Folorunso SA Chukwu AU Tongo O. Prevalence and factors associated with neonatal jaundice: a case study of University College Hospital, Ibadan. J Dent Med Sci (IOSR-JDMS). 2015;14:17-23.

[17] Devi GVR Bhuvaneswari M Prasad GR Clinical profile and outcome of term and preterm newborns with hyperbilirubinemia admitted in SNCU of a teaching hospital. J Evid Based Med Healthc. 2015;2:2228-2236.

[18] Palmer DC Drew JH. Jaundice: a 10 year review of 41,000 live born infants. J Paediatr Child Health. 1983;19:86-89. 10.1111/j.1440-1754.1983.tb02063.x 6414449

[19] Muhe LM McClure EM Nigussie AK, et al Major causes of death in preterm infants in selected hospitals in Ethiopia (SIP): a prospective, cross-sectional, observational study. Lancet Glob Health. 2019;7:e1130-e1138. 31303299 10.1016/S2214-109X(19)30220-7 PMC6639243

[20] Chawla D Jain S Dhir S Rani S. Risk assessment strategy for prediction of pathological hyperbilirubinemia in neonates. Indian J Pediatr. 2012;79:198-201. 21541649 10.1007/s12098-011-0409-x

[21] Kaur S Chawla D Pathak U Jain S. Predischarge non-invasive risk assessment for prediction of significant hyperbilirubinemia in term and late preterm neonates. J Perinatol. 2012;32:716-721. 22094493 10.1038/jp.2011.170

[22] Crosse VM Meyer TC Gerrard JW. Kernicterus and prematurity. Arch Dis Child. 1955;30:501-508. 13275980 10.1136/adc.30.154.501 PMC2011823

[23] Oh W Tyson JE Fanaroff AA, et al Association between peak serum bilirubin and neurodevelopmental outcomes in extremely low birth weight infants. Pediatrics. 2003;112:773-779. 14523165 10.1542/peds.112.4.773

[24] Morris BH Oh W Tyson JE, et al Aggressive vs. conservative phototherapy for infants with extremely low birth weight. N Engl J Med. 2008;359:1885-1896. 18971491 10.1056/NEJMoa0803024 PMC2821221

[25] Stevenson DK Wong RJ Arnold CC Pedroza C Tyson JE. Phototherapy and the risk of photo-oxidative injury in extremely low birth weight infants. Clin Perinatol. 2016;43:291-295. 27235208 10.1016/j.clp.2016.01.005

[26] Arif K Bhutta ZA. Risk factors and spectrum of neonatal jaundice in a birth cohort in Karachi. Indian Pediatr. 1999;36:487-493. 10728039

[27] Fekete M Horváth M Vincellér M. Perinatal asphyxia and jaundice in newborn infants. Acta Paediatr Acad Sci Hung. 1978;19:17-26. 665220

[28] Ochigbo SO Venn I Anachuna K. Prevalence of bilirubin encephalopathy in Calabar, South-South Nigeria: a five-year review study. Iran J Neonatol. 2016;7:9-12.

[29] Wei K-L Yang Y-J Yao Y-J, et al Epidemiologic survey on hospitalized neonates in China. Transl Pediatr. 2012;1:15-22. 26835259 10.3978/j.issn.2224-4336.2011.10.01 PMC4728849

[30] Gamaleldin R Iskander I Seoud I, et al Risk factors for neurotoxicity in newborns with severe neonatal hyperbilirubinemia. Pediatrics. 2011;128:e925-e931. 21911352 10.1542/peds.2011-0206 PMC3182847

[31] Tiwari PK Bhutada A Agarwal R Basu S Raman R Kumar A. UGT1A1 gene variants and clinical risk factors modulate hyperbilirubinemia risk in newborns. J Perinatol. 2014;34:120-124. 24232666 10.1038/jp.2013.140

